# Gaps in Emergency General Surgery Coverage in the United States

**DOI:** 10.1097/AS9.0000000000000043

**Published:** 2021-02-18

**Authors:** Angela M. Ingraham, Scott M. Chaffee, M. Didem Ayturk, Victor K. Heh, Catarina I. Kiefe, Heena P. Santry

**Affiliations:** From the * Department of Surgery, University of Wisconsin, Madison, WI; † Department of Surgery, The Ohio State University, Columbus, OH; ‡ Center for Surgical Health Assessment, Research, and Policy (SHARP), The Ohio State University, Columbus, OH; §Department of Population and Quantitative Health Sciences, University of Massachusetts Medical School, Worcester, MA.

## Abstract

**Introduction:**

Despite 3 million adults in the United States being admitted annually for emergency general surgery (EGS) conditions, which disproportionately affect vulnerable populations, we lack an understanding of the barriers to round-the-clock EGS care. Our objective was to measure gaps in round-the-clock EGS care.

**Methods:**

From August 2015 to December 2015, we surveyed all US-based adult acute care general hospitals that have an emergency room and ≥1 operating room and provided EGS care, utilizing paper and electronic methods. Surgeons or chief medical officers were queried regarding EGS practices.

**Results:**

Of 2811 hospitals, 1634 (58.1%) responded; 279 (17.1%) were unable to always provide round-the-clock EGS care. Rural location, smaller bed size, and nonteaching status were associated with lack of round-the-clock care. Inconsistent surgeon coverage was the primary reason for lacking round-the-clock EGS care (n = 162; 58.1%). However, lack of a tiered system for booking emergency cases, no anesthesia availability overnight, and no stipend for EGS call were also associated with the inability to provide round-the-clock EGS care.

**Discussion:**

We found significant gaps in access to EGS care, often attributable to workforce deficiencies.

## Abstract 2

**Mini-abstract:** Gaps in Emergency General Surgery Coverage in the United States is a national survey-based research study that highlights the presence of significant gaps in emergency general surgery (EGS) care. The surveyed population was determined using the 2013 American Hospital Association Annual Survey of US hospitals to identify acute care general hospital accessible to the general public and presence of an emergency room and at least one operating room. Survey results indicate gaps in round-the-clock EGS care exist in the United States. The declining general surgery workforce presents a substantial reason for hospitals’ inability to provide round-the-clock EGS care.

Supplemental Digital Content is available in the text.

## INTRODUCTION

Emergency general surgery (EGS) care is an under-recognized, yet significant public health burden. EGS conditions afflict more than 12,900 of 1,000,000 persons annually, exceeding that of common, highly publicized, and well-studied public health concerns, such as new-onset diabetes mellitus (899 of 1,000,000 persons annually) and newly diagnosed cancers (650 of 1,000,000 persons annually).^1–4^ EGS patients constitute 7.2% of total hospitalizations in the United States, with more than a quarter of these patients requiring surgery during their admission.^1^ The number of EGS patients is growing with a 27% increase over 10 years, from 6.4% in 2001 to 7.8% in 2010.^1^ Additionally, the total national cost of EGS hospitalizations in 2010, not including physician fees or posthospitalization care, was approximately $28.4 billion. With the increasing number and aging of the US population, this cost is expected to rise to more than $41 billion by 2060. Thus, EGS care ranks as the most expensive cause of unplanned hospitalization in the United States.^5^

In addition to the burden of disease, EGS patients are at high risk for morbidity and mortality. Approximately half of all patients undergoing EGS will develop a postoperative complication.^2^ Additionally, between 4% and 14% will be readmitted to the hospital within 30 days of their surgery for a skin/soft-tissue or intrabdominal diagnosis.^3^ While EGS patients are complex, with half being over the age of 60 and most having comorbidities,^1^ EGS is an independent predictor of poorer outcomes. In a study using American College of Surgeons National Surgical Quality Improvement Program (ACS NSQIP) data, after controlling for the significantly higher number of comorbid conditions based on ACS-NSQIP preoperative risk assessment, EGS patients were 39% more likely to die within 30 days EGS patients than their counterparts undergoing the same procedures electively.^2^

While EGS care treats the most acutely ill, highest risk, and most costly general surgery patients,^1–4^ ensuring their access to that care is a looming public health crisis. The ever-increasing and aging US population is not being matched by graduating general surgeons.^6^ The number of general surgeons in practice in the United States decreased by 2.3% between 1996 and 2006. Increased subspecialization, lifestyle demands, early retirement, and reimbursement pressures have led to fewer surgeons providing emergency coverage.^7–13^ Williams et al projected a 9.2% decrease in general surgeons per capita from 2010 to 2030.^9^ Counties with greater percentages of black, Hispanic, uninsured, and low-education individuals as well as rural counties disproportionately lack access to EGS care.^14^

In this study, we specifically sought to understand the barriers to round-the-clock EGS care at the hospital level. Thus, the objective of this study was to measure gaps in round-the-clock EGS care in the United States and determine hospital-level predictors of insufficient EGS care.

## METHODS

### Survey Methodology

We conducted a national survey to determine variations in the delivery of EGS care across all US hospitals where an adult with a general surgery emergency might seek care. Our methods including identification of hospitals providing EGS care, identification of questionnaire respondents (95% surgeons, 4.7% chief medical officers at locations where there was only a single general surgery who did not respond), questionnaire development using an iterative mixed methods process, and our hybrid paper/electronic survey implementation between August and December 2015 conducted in 2 waves have been described elsewhere.^15,16^ A copy of the questionnaire can be found in Appendix 1, http://links.lww.com/AOSO/A19.

Three questions in the survey focused on round-the-clock EGS care. The first asked “Does your hospital ever *lack* round-the-clock (24/7/365) emergency general surgery coverage?” This yes/no question included a drop sequence for those responding yes to further answer “Approximately how often does your hospital *lack* emergency general surgery coverage?” (answered numerically as a percentage) and “How frequent are the following reasons for *lacking* coverage?” (answered for “Lack of general surgery coverage,” “Lack of anesthesia coverage,” “Lack of OR staff,” “Emergency room is on diversion,” and “Other (please specify) ________” using a Likert scale of Always, Often, Sometimes, Rarely, and Never).

By the end of 2 unique survey implementations, there were 1690 responses from the 2811 eligible acute care hospitals, representing a total response rate of 60.1%. Of these 1634 hospitals responded to our questions specifically regarding round-the-clock EGS coverage, representing 58.1% of the initial 2811 hospitals surveyed. In this manuscript, we present results that are related to the ability of the hospital to provide round-the-clock EGS care. Specific practices related to the structure of EGS teams, ancillary hospital services, and human resources are addressed in other manuscripts.

### Statistical Analysis

Questionnaire responses on the availability of round-the-clock EGS care were tabulated and compared by hospital characteristics (geographic region, ownership type, hospital location, teaching status, inpatient bed capacity, medical school affiliation, and trauma certification as reported by the AHA in 2015) using univariate comparisons (χ^2^). The independent association of any given hospital characteristic with the availability of round-the-clock EGS care was determined using multivariable logistic regression. Hospital characteristics found to have *P* < 0.20 in univariate comparisons were included in the model. A similar model was constructed for operating room access variables, as reported in the survey (and number of operating rooms as reported in the AHA data), to determine which structures and processes were associated with lack of round-the-clock EGS care. These variables were not included in single model with hospital characteristics due to multicollinearity. Referent categories for structure and process variables were chosen based on prior data regarding which operating room structure and process variables were generally consistent with the acute care surgery model of care designed to provide urgent, round-the-clock access to EGS.^17^

Maps were generated using Maptitude GIS Mapping Software (Caliper Corp, 2016). All analyses were performed using SAS 9.4 (SAS Institute, Cary, NC). This study was reviewed and deemed exempt by the senior authors’ Institutional Review Board.

## RESULTS

**FIGURE 1.**
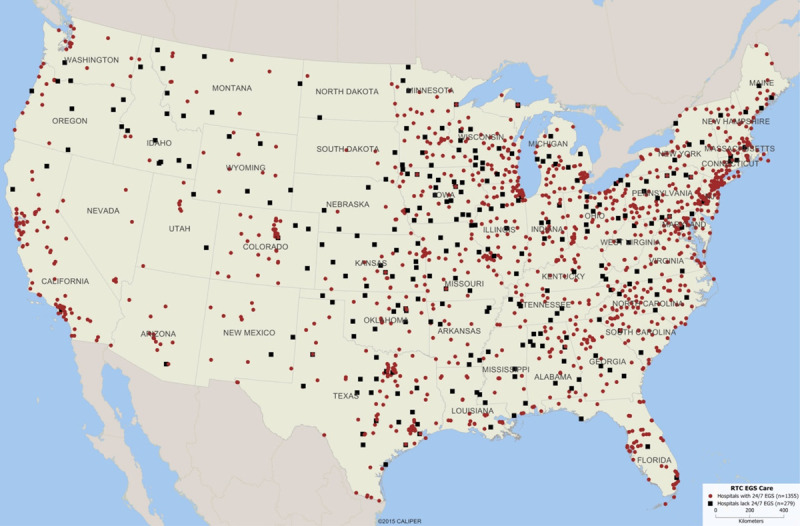
Map of 1,634 US-based, adult acute care general hospitals that have an emergency room and ≥1 operating room that do (circle; n = 1355) and do not (square; n = 279) provide round-the-clock EGS care. EGS indicates emergency general surgery.

**FIGURE 2.**
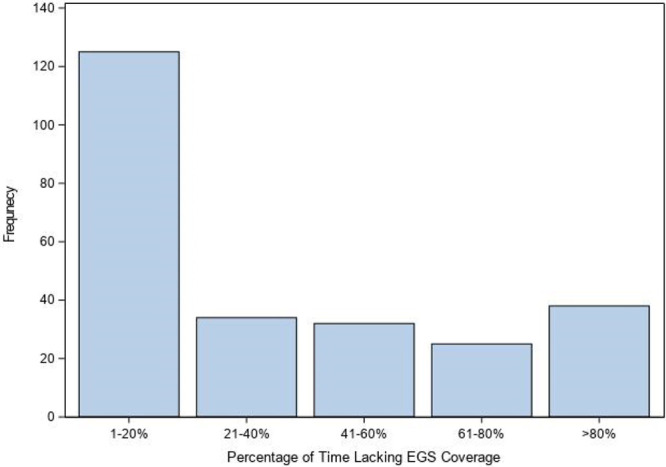
Histogram of estimated percentage of time hospitals that do not provide round-the-clock emergency general surgery care are not able to provide it (n = 279). Percentage of time by self-report ranging from 1 to 99. EGS indicates emergency general surgery.

Characteristics of hospitals that did and did not respond to our query are detailed in Appendix 2, http://links.lww.com/AOSO/A20.^18^ Of the 1634 hospitals that responded to our query about round-the-clock EGS care, 279 (17.1%) hospitals lacked round-the-clock EGS care on average 35.7% (SD = 33.6) of the time. Figure 1 depicts the locations across the United States of hospitals that do provide (circles; n = 1355) and do not provide (squares; n = 279) round-the-clock EGS care. Figure 2 depicts distribution of the self-reported percentage of time the latter hospitals lack EGS coverage. A descriptive summary of hospital characteristics comparing those that do and do not provide round-the-clock EGS care is provided in Table 1. Hospitals that do not provide round-the-clock EGS care were more likely to have non-governmental ownership, be located in rural areas, not have teaching affiliations, not be affiliated with a medical school, have smaller bed-sizes, be located in the West North Central region, and not have trauma certification. The table in Appendix 3, http://links.lww.com/AOSO/A21, shows the association of these same characteristics with amount of time EGS coverage was not available.

**TABLE 1.** Characteristics Among Acute Care General Hospitals in the United States That Do or Do Not Provide Round-the-Clock Emergency General Surgery Care

Variable	Provide Round-the-Clock EGS Care (n = 1355), n (%)	Do Not Provide Round-the-Clock EGS Care (n = 279), n (%)	*p*
**Ownership**			<0.0001
Nongovernmental	990 (73.1)	153 (54.8)	
Governmental (nonfederal)	217 (16.0)	88 (31.5)	
Investor-owned	148 (10.9)	37 (13.3)	
**Location**			<0.0001
Urban	969 (71.5)	77 (27.6)	
Rural	386 (28.5)	202 (72.4)	
**Teaching status**			<0.0001
Major	158 (11.7)	2 (0.7)	
Minor	568 (41.9)	43 (15.4)	
Nonteaching	629 (46.4)	234 (83.9)	
**Medical school affiliation**			<0.0001
Yes	551 (40.7)	30 (10.8)	
No	804 (59.3)	249 (89.2)	
**Bed size**			<0.0001
<100	393 (29.0)	242 (86.7)	
100–199	347 (25.6)	24 (8.6)	
200–299	215 (15.9)	8 (2.9)	
300–399	146 (10.8)	2 (0.7)	
400 or more beds	254 (18.7)	3 (1.1)	
**Region**			<0.0001
New England	81 (6.0)	9 (3.2)	
East North Central	237 (17.5)	50 (17.9)	
East South Central	92 (6.8)	31 (11.1)	
Middle Atlantic	178 (13.1)	13 (4.7)	
Mountain	99 (7.3)	30 (10.8)	
Pacific	129 (9.5)	12 (4.3)	
South Atlantic	236 (17.4)	29 (10.4)	
West North Central	152 (11.2)	62 (22.2)	
West South Central	151 (11.1)	43 (15.4)	
**Trauma certification**			0.0043
Yes	675 (49.8)	112 (40.1)	
No	608 (44.9)	149 (53.4)	

The sample size was n = 1634. Cell totals may not equal 1634 if the variable was missing or not reported in the American Hospital Association Annual Survey.

Among the 279 hospitals that responded that they could not provide round-the-clock EGS care, reasons for being unable to provide such care varied (Table 2). The most common reason for not being able to provide round-the-clock EGS care was a lack of general surgery coverage with 162 (58.1%) of the 279 hospitals citing this as the reason they could not provide round-the-clock EGS care “Always” or “Often” whereas lack of anesthesia coverage (55 [19.7%]), lack of operating room staff (54 [19.4%]), and the emergency room being on diversion (6 [2.1%]) were less often cited.

**TABLE 2.** Reasons for Not Providing Round-the-Clock Emergency General Surgery Care at Acute Care General Hospitals in the United States

Response	Lack of General Surgery Coverage, n (%)	Lack of Anesthesia Coverage, n (%)	Lack of Operating Room Staff, n (%)	Emergency Room Is on Diversion, n (%)
**Always**	113 (40.5)	33 (11.8)	32 (11.5)	4 (1.4)
**Often**	49 (17.6)	22 (7.9)	22 (7.9)	2 (0.7)
**Sometimes**	52 (18.6)	26 (9.3)	25 (9.0)	27 (9.7)
**Rarely**	52 (18.6)	51 (18.3)	49 (17.6)	113 (40.5)
**Never**	11 (3.9)	142 (50.9)	144 (51.6)	126 (45.2)
**Missing**	2 (0.7)	5 (0.4)	7 (0.5)	7 (2.5)

The sample size was n = 279.

Table 3 compares hospital that did and did not provide round-the-clock EGS care based on survey responses regarding operating room access. Hospitals with fewer operating rooms, less block time, lack of a tiered system for booking emergency cases or a process to defer elective cases, higher frequency of surgeons covering EGS call having competing clinical roles, working postcall, not taking in-house call, not also covering trauma or ICU while on EGS call, and not receiving a stipend for taking EGS call were associated with lack of providing round-the-clock EGS care. Lower rates of in-house or on-call perioperative staff were also associated with lack of providing round-the-clock EGS care.

**TABLE 3.** Differences in Structure and Processes as They Relate to Operating Room Access Between Hospitals That Do or Do Not Provide Round-the-Clock EGS Care

	Provide Round-the-Clock EGS Care (n = 1355), n (%)	Do Not Provide Round-the-Clock EGS Care (n = 279), n (%)	p *
**Operating room availability**			
Number of operating rooms			
<10	646 (47.7%)	247 (86.4%)	<0.0001
10–20	362 (26.7%)	9 (3.2%)	
>20	247 (18.2%)	4 (1.4%)	
Not available	100 (7.4%)	25 (9.0%)	
Block time for EGS (%)			
<1 block time	1065 (78.6%)	249 (89.3%)	<0.0001
1–4 d	61 (4.5%)	12 (4.3%)	
≥5 d	187 (13.8%)	5 (1.8%)	
Not available	42 (3.1%)	13 (4.7%)	
Tiered system for booking emergent surgical cases (yes)	912 (67.3%)	110 (39.4%)	<0.0001
Process to defer elective cases (yes)	977 (72.1%)	151 (54.1%)	<0.0001
**Surgical coverage**			
Daytime surgeons covering EGS free of other clinical duties (yes)	169 (12.5%)	6 (2.2%)	<0.0001
Daytime surgeon on call for EGS working postcall			
Always/often	1095 (80.8%)	240 (89.0%)	<0.0001
Sometimes	129 (9.5%)	3 (1.1%)	
Rarely/never	89 (6.6%)	4 (1.4%)	
In-house surgeon overnight for EGS			<0.0001
Always/often	458 (33.8%)	52 (18.6%)	
Sometimes	87 (6.4%)	18 (6.5%)	
Rarely/never	766 (56.5%)	175 (62.7%)	
Overnight surgeon also responsible for covering trauma			
Always/often	859 (63.4%)	137 (49.1%)	<0.0001
Sometimes	78 (5.8%)	22 (7.9%)	
Rarely/never	376 (27.8%)	88 (31.4%)	
Overnight surgeon also responsible for covering ICU care			
Always/often	457 (33.7%)	56 (20.1%)	<0.0001
Sometimes	148 (10.9%)	19 (6.8%)	
Rarely/never	708 (52.3%)	173 (62.0%)	
Overnight surgeon also responsible for covering EGS at more than one hospital			
Always/often	223 (16.5%)	39 (14.0%)	<0.0001
Sometimes	159 (11.7%)	25 (9.0%)	
Rarely/never	929 (68.6%)	181 (64.9%)	
Surgeon covering EGS receives stipend beyond billing for services rendered			<0.0001
Always/often	537 (39.6%)	37 (13.3%)	
Sometimes	93 (6.9%)	17 (6.1%)	
Rarely/never	677 (50.0%)	193 (69.2%)	
**Overnight perioperative staffing**			
Overnight scrub techs			
None	4 (0.3%)	1 (0.4%)	<0.0001
On-call	929 (68.6%)	194 (69.5%)	
In-house	390 (28.8%)	6 (2.2%)	
Overnight OR nurses			<0.0001
None	–	–	
On-call	938 (69.2%)	196 (70.3%)	
In-house	390 (28.8)	5 (1.8%)	
Overnight recovery room nurses			<0.0001
None	24 (1.8%)	11 (3.9%)	
On-call	1091 (80.5%)	186 (66.7%)	
In-house	208 (15.4%)	3 (1.1%)	
Overnight anesthesia staff (MD, DO, CRNA)			
None	149 (11.0%)	122 (43.7%)	<0.0001
On-call	732 (54.0%)	57 (20.4%)	
In-house	432 (31.9%)	9 (3.2%)	

*Student *t* test, Wilcox Rank Sum, and χ^2^ tests of association where appropriate.

EGS indicates emergency general surgery.

Among the 1634 hospitals who responded to our query on whether they do or do not provide round-the-clock EGS care, on multivariable analysis for hospital characteristics, governmentally owned hospitals, hospitals in rural locations, hospitals with <100 beds, as well as hospitals in the East South Central, West North Central, and West South Central regions had higher odds of lacking round-the-clock EGS care as detailed in (Table 4). Finally, in multivariable analysis of structures and processes that facilitate operating room access, only lack of a tiered system for booking emergency cases, no anesthesia availability overnight, and no stipend for EGS call were associated with inability to provide round-the-clock EGS care (Table 5).

**TABLE 4.** Predictors of Inability to Provide Round-the-Clock Emergency General Surgery Coverage Among Acute Care General Hospitals in the United States

Variable	OR (95% CI)
**Ownership**	
Nongovernmental	Reference
Governmental (nonfederal)	1.5 (1.0–2.2)
Investor-owned	1.5 (0.9–2.6)
**Location**	
Urban	Reference
Rural	1.8 (1.3–2.6)
**Teaching status**	
Major	Reference
Minor	0.9 (0.2–5.5)
Nonteaching	1.3 (0.2–8.9)
**Medical school affiliation**	
Yes	Reference
No	1.0 (0.5–2.0)
**Bed size**	
400 or more beds	Reference
<100	22.3 (5.1–98.8)
100–199	3.2 (0.7–14.2)
200–299	2.3 (0.5–11.0)
300–399	1.0 (0.2–7.0)
**Region**	
New England	Reference
East North Central	1.6 (0.7–3.8)
East South Central	3.4 (1.3–8.9)
Middle Atlantic	1.7 (0.6–4.6)
Mountain	2.4 (1.0–6.2)
Pacific	1.2 (0.4–3.4)
South Atlantic	1.2 (0.5–3.0)
West North Central	2.5 (1.0–5.9)
West South Central	2.7 (1.1–6.9)
**Trauma certification**	
Yes	Reference
No	1.3 (0.9–1.8)

The sample size was n = 1634.

CI indicates confidence interval; OR, odds ratio.

**TABLE 5.** Operating Room Structure and Process Predictors of Inability to Provide Round-the-Clock Emergency General Surgery Coverage Among Acute Care General Hospitals in the United States

	OR (95% CI)
**Operating room availability**	
Number of operating rooms	
<10	1.85 (0.49–6.98)
10–20	0.66 (0.17–2.61)
>20	Ref
Block time for EGS (%)	
<1 block time	2.64 (0.82–8.51)
1–4 d	4.42 (0.98–19.97)
>5 d	Ref
Tiered system for booking emergent surgical cases	
Yes	Ref
No	3.37 (1.87–6.07)
Process to defer elective cases (yes)	
Yes	Ref
No	0.64 (0.35–1.19)
**Surgical coverage**	
Daytime surgeons covering EGS free of other clinical duties	
* *Yes	Ref
* *No	1.13 (0.36–3.58)
Daytime surgeon on call for EGS working postcall	
Always/often	0.91 (0.14–5.81)
Sometimes	0.51 (0.06–4.38)
Rarely/never	Ref
In-house surgeon overnight for EGS	
Always/often	Ref
Sometimes	1.61 (0.66–3.95)
Rarely/never	0.83 (0.49–1.39)
Overnight surgeon also responsible for covering trauma	
Always/often	Ref
Sometimes	2.8 (1.18–6.67)
Rarely/never	1.22 (0.71–2.11)
Overnight surgeon also responsible for covering ICU care	
Always/often	Ref
Sometimes	0.62 (0.27–1.46)
Rarely/never	1.01 (0.60–1.68)
Overnight surgeon also responsible for covering EGS at more than one hospital	
Always/often	Ref
Sometimes	2.29 (0.98–5.33)
Rarely/never	1.47 (0.79–2.75)
Surgeon covering EGS receives stipend beyond billing for services rendered	
Always/often	Ref
Sometimes	1.54 (0.58–4.12)
Rarely/never	2.84 (1.67–4.87)
**Overnight perioperative staffing**	
Overnight scrub techs	
None	–
On-call	0.64 (0.13–3.11)
In-house	Ref
Overnight OR nurses	
None	–
On-call	2.90 (0.49–17.14)
In-house	Ref
Overnight recovery room nurses	
None	0.74 (0.10–5.41)
On-call	0.75 (0.13–4.23)
In-house	Ref
Overnight anesthesia staff (MD, DO, CRNA)	
None	16.78 (5.65–49.81)
On-call	1.88 (0.67–5.25)
In-house	Ref

CI indicates confidence intervals; EGS, emergency general surgery; OR, odds ratio.

## DISCUSSION

In this novel, survey of hospitals on the details of how EGS care is provided across the United States, we sought to quantify gaps in round-the-clock EGS care and determine hospital-level predictors of insufficient EGS care. We found that significant gaps in access to round-the-clock EGS care exist in the United States. These gaps are often attributable to workforce deficiencies, particularly a lack of general surgeons, and are primarily at small or rural hospitals. We also describe several factors related to operating room access (operating room availability, surgical coverage, and overnight perioperative staffing). Importantly, in addition to surgeon availability, the structure implemented to tier cases by urgency, the availability of anesthesia staff, and the compensation of general surgeons were associated with lack of round-the-clock EGS care. This emphasizes the fact that emergency general surgery care must be guided and supported by public health efforts and policies.

In 2006, the Institute of Medicine described emergency care as being at the “breaking point” in the United States.^19^ EGS is a crucial component of emergency care. The public health crisis in delivering EGS care is being accelerated by an imbalance between patients in need and providers to meet the demand.^20^ While availability and access are declining due to a decrease in the physician workforce^8^ and an increase in emergency department closures,^21^ the volume of patients with EGS conditions is increasing. The number of Americans with EGS conditions rose from 2.4 million in 2001 to 3.0 million in 2010.^1,22,23^ The annual incidence of EGS conditions in the United States (1290 cases/100,000) approaches that of acute myocardial infarction (1462/100,000) and cerebrovascular accident (1472/100,000), 2 diseases that warrant comparable access to care for prompt diagnosis and treatment.^1,24^

As with myocardial infarction and stroke, EGS patients are particularly vulnerable to crises in emergency care as these conditions are associated with high morbidity and mortality and require round-the-clock access to care. Unlike our study, studies of the imbalance between patients’ needs and access to EGS care have previously focused on patient-level factors, such as insurance coverage,^25,26^ or surgeon-level factors, such as the reasons for the decreased number of general surgeons in the United States.^10^

A 2010 survey of emergency room directors at 715 hospitals nationally found that 37% reported inadequate EGS coverage.^27^ In the present survey, largely of surgeons responsible for overseeing EGS coverage, we found that 17% struggled with providing round-the-clock EGS care. There are several possible reasons for this discrepancy. First, our hospital characteristics were different. Hospitals represented in our survey responses were more likely than those in the survey by Rao et al to be nongovernmental in ownership and less likely to be in the South while more likely to be in the Midwest.^27^ Additionally, surgeons may feel that by having a surgeon listed on the “on-call schedule” that they are providing round-the-clock EGS coverage. However, we recently reported that 11% of surgeons taking EGS call always/often provide EGS care at more than 1 hospital, in essence reducing availability in certain circumstances.^28^ Unfortunately, when a surgeon is not available, care must either be delayed or the patient must be transferred elsewhere for definitive diagnosis and treatment. One study examining such transfers found that rural residents were often transferred for common procedures, such as inguinal hernia repairs and cholecystectomies, and traveled an average of 67 miles for care.^29^ Yet, these transfers might be necessary even if a surgeon capable of performing the necessary operation is available, for example, due to need for specialty critical care resources in the case of patients with significant cardiopulmonary comorbidities but otherwise straightforward surgical problem (eg, appendicitis) or need subspecialty nonsurgical care not available locally (eg, ERCP in the case of choledocholithiasis. Importantly, however, at many referral centers, surgeons covering EGS also cover ICU and trauma^28^; these competing interests may further delay access to care based on acuity and availability of back-up surgeons.

Compared with other medical and surgical subspecialties, such as cardiac, stroke, and trauma care, providers, hospital and healthcare administrators, as well as public health and policy officials lag behind in efforts to improve the care provided to EGS patients. To the best of our knowledge, this is the most comprehensive study of barriers to round-the-clock EGS care nationally. Given the existing imbalance between the needs of EGS patients and the surgical workforce in the United States, a regionalized system drawing upon the lessons learned from stroke,^30^ neonatal intensive,^31^ acute coronary syndrome,^32^ as well as trauma^33^ care may optimize access and quality for EGS patients. While it is not sustainable for all EGS conditions to be transferred to a higher level of care from patient, provider, or healthcare system vantage points, strategic planning involving thoughtful allocation of limited resources and deliberate transfers of patients to tertiary centers with round-the-clock general surgery capabilities should be considered to ensure adequate access to EGS care nationally.

### Limitations

While a 58% response rate is laudable for survey research, especially among physicians,^34^ 42% of hospitals where an adult with an EGS condition might seek care were not represented in our study. Our comparison of responders to nonresponders showed that responders were more likely to represent large, nonprofit hospitals with a teaching affiliation (Appendix 2, http://links.lww.com/AOSO/A20); therefore, our results may be less generalizable to smaller, governmental, or for-profit hospitals without a teaching affiliation. Second, a limitation of any survey is that the information is only as reliable as the individual who is completing the survey. Targeted efforts were made to ensure that the individual completing the survey was the individual most knowledgeable of the care provided to EGS patients at his/her respective institution.

## CONCLUSION

We document that significant gaps in round-the-clock EGS care exist in the United States. A substantial reason for the inability of hospitals to provide care is a lack of general surgeons. While the declining general surgery workforce has been a subject of much scrutiny for at least 2 decades,^8–11,35^ to our knowledge, this is the first comprehensive national assessment of gaps in EGS care from a hospital perspective. Our results serve to inform policy and performance improvement efforts to ensure that all Americans have timely, appropriate access to EGS care.

## Supplementary Material

## References

[1] Gale SC Shafi S Dombrovskiy VY. The public health burden of emergency general surgery in the United States: a 10-year analysis of the nationwide inpatient sample–2001 to 2010. J Trauma Acute Care Surg. 2014; 77:202–208 25058242 10.1097/TA.0000000000000362

[2] Havens JM Peetz AB Do WS. The excess morbidity and mortality of emergency general surgery. J Trauma Acute Care Surg. 2015; 78:306–311 25757115 10.1097/TA.0000000000000517

[3] Havens JM Olufajo OA Cooper ZR. Defining rates and risk factors for readmissions following emergency general surgery. JAMA Surg. 2016; 151:330–336 26559368 10.1001/jamasurg.2015.4056

[4] Kassin MT Owen RM Perez SD. Risk factors for 30-day hospital readmission among general surgery patients. J Am Coll Surg. 2012; 215:322–330 22726893 10.1016/j.jamcollsurg.2012.05.024 PMC3423490

[5] Ogola GO Gale SC Haider A. The financial burden of emergency general surgery: national estimates 2010 to 2060. J Trauma Acute Care Surg. 2015; 79:444–448 26307879 10.1097/TA.0000000000000787

[6] Voelker R. Experts say projected surgeon shortage a “looming crisis” for patient care. JAMA. 2009; 302:1520–1521 19826016 10.1001/jama.2009.1456

[7] Rudkin SE Oman J Langdorf MI. The state of ED on-call coverage in California. Am J Emerg Med. 2004; 22:575–581 15666264 10.1016/j.ajem.2004.08.001

[8] Cofer JB Burns RP. The developing crisis in the national general surgery workforce. J Am Coll Surg. 2008; 206:790–795 18471697 10.1016/j.jamcollsurg.2007.12.017

[9] Williams TE Jr Satiani B Thomas A. The impending shortage and the estimated cost of training the future surgical workforce. Ann Surg. 2009; 250:590–597 19730238 10.1097/SLA.0b013e3181b6c90b

[10] Etzioni DA Finlayson SR Ricketts TC. Getting the science right on the surgeon workforce issue. Arch Surg. 2011; 146:381–384 21502445 10.1001/archsurg.2011.64

[11] ACS Health Policy Research Institute and the American Association of Medical Colleges. *The Surgical Workforce in the United States*: Profile and Recent Trends. American College of Surgeons (ACS) Health Policy Research Institute;. 2010

[12] Hutter MM. Specialization: the answer or the problem? Ann Surg. 2009; 249:717–718 19387302 10.1097/01.sla.0000348651.75237.df

[13] Borman KR Vick LR Biester TW. Changing demographics of residents choosing fellowships: longterm data from the American Board of Surgery. J Am Coll Surg. 2008; 206:782–788 18471695 10.1016/j.jamcollsurg.2007.12.012

[14] Khubchandani JA Shen C Ayturk D. Disparities in access to emergency general surgery care in the United States. Surgery. 2018; 163:243–250 29050886 10.1016/j.surg.2017.07.026 PMC6071308

[15] Santry HP Strassels SA Ingraham AM. Identifying the fundamental structures and processes of care contributing to emergency general surgery quality using a mixed-methods Donabedian approach. BMC Med Res Methodol. 2020; 20:247 33008294 10.1186/s12874-020-01096-7 PMC7532630

[16] Ingraham AM Ayturk MD Kiefe CI. Adherence to 20 emergency general surgery best practices: results of a national survey. Ann Surg. 2019; 270:270–280 29608545 10.1097/SLA.0000000000002746

[17] Ricci KB Rushing AP Ingraham AM. The association between self-declared acute care surgery services and operating room access: results from a national survey. J Trauma Acute Care Surg. 2019; 87:898–906 31205221 10.1097/TA.0000000000002394 PMC8278363

[18] Khubchandani JA Ingraham AM Daniel VT. Geographic diffusion and implementation of acute care surgery: an uneven solution to the National Emergency General Surgery Crisis. JAMA Surg. 2018; 153:150–159 28979986 10.1001/jamasurg.2017.3799 PMC5838713

[19] Institute of Medicine. Hospital-Based Emergency Care: At the Breaking Point. 2007, The National Academies Press. doi: 10.17226/11621

[20] Kellermann AL. Crisis in the emergency department. N Engl J Med. 2006; 355:1300–1303 17005946 10.1056/NEJMp068194

[21] Hsia RY Kellermann AL Shen YC. Factors associated with closures of emergency departments in the United States. JAMA. 2011; 305:1978–1985 21586713 10.1001/jama.2011.620 PMC4063529

[22] Liu JH Etzioni DA O’Connell JB. The increasing workload of general surgery. Arch Surg. 2004; 139:423–428 15078711 10.1001/archsurg.139.4.423

[23] Etzioni DA Liu JH Maggard MA. The aging population and its impact on the surgery workforce. Ann Surg. 2003; 238:170–177 12894008 10.1097/01.SLA.0000081085.98792.3d PMC1422682

[24] Benjamin EJ Blaha MJ Chiuve SE; American Heart Association Statistics Committee and Stroke Statistics Subcommittee. Heart disease and stroke statistics-2017 update: a report from the American Heart Association. Circulation. 2017; 135:e146–e603 28122885 10.1161/CIR.0000000000000485 PMC5408160

[25] Scott JW Havens JM Wolf LL. Insurance status is associated with complex presentation among emergency general surgery patients. Surgery. 2017; 161:320–328 27712875 10.1016/j.surg.2016.08.038

[26] Ho VP Nash GM Feldman EN. Insurance but not race is associated with diverticulitis mortality in a statewide database. Dis Colon Rectum. 2011; 54:559–565 21471756 10.1007/DCR.0b013e31820d188f

[27] Rao MB Lerro C Gross CP. The shortage of on-call surgical specialist coverage: a national survey of emergency department directors. Acad Emerg Med. 2010; 17:1374–1382 21091822 10.1111/j.1553-2712.2010.00927.x

[28] Daniel VT Rushing A Ingraham A. Association Between Enhanced Oveernight Operating Room Access and Mortality for True Life-Threatening Surgical Disease. 2019, Eastern Association for the Surgery of Trauma 10.1097/TA.0000000000002267 PMC6921508 31242499

[29] Misercola B Sihler K Douglas M. Transfer of acute care surgery patients in a rural state: a concerning trend. J Surg Res. 2016; 206:168–174 27916358 10.1016/j.jss.2016.06.090

[30] Xian Y Holloway RG Chan PS. Association between stroke center hospitalization for acute ischemic stroke and mortality. JAMA. 2011; 305:373–380 21266684 10.1001/jama.2011.22 PMC3290863

[31] American Academy of Pediatrics Committee on F, Newborn. Levels of neonatal care. Pediatrics. 2012; 130:587–597 22926177 10.1542/peds.2012-1999

[32] Westfall JM Kiefe CI Weissman NW. Does interhospital transfer improve outcome of acute myocardial infarction? A propensity score analysis from the Cardiovascular Cooperative Project. BMC Cardiovasc Disord. 2008; 8:22 18782452 10.1186/1471-2261-8-22 PMC2551582

[33] Nathens AB Brunet FP Maier RV. Development of trauma systems and effect on outcomes after injury. Lancet. 2004; 363:1794–1801 15172780 10.1016/S0140-6736(04)16307-1

[34] VanGeest JB Johnson TP Welch VL. Methodologies for improving response rates in surveys of physicians: a systematic review. Eval Health Prof. 2007; 30:303–321 17986667 10.1177/0163278707307899

[35] Fischer JE. The impending disappearance of the general surgeon. JAMA. 2007; 298:2191–2193 18000204 10.1001/jama.298.18.2191

