# Supplementary material for: Gaps in Emergency General Surgery Coverage in the United States
Source: Ann Surg Open. 2021 Feb 18;2(1):e043. doi: 10.1097/AS9.0000000000000043 (PMC8409136; doi:10.1097/AS9.0000000000000043)
Supplement: Supplementary file 2 [file as9-2-e043-s002.pdf]

APPENDIX 2 - Characteristics of Responded Hospitals versus Non-Responded Hospitals

	Non-Responded Hospitals (n=1,121), n (%)	Responded Hospitals (n=1,690), n (%)	p-value
**Ownership**			<.0001
Non-Governmental	639 (57)	1199 (70.9)	
Governmental. (non-federal)	268 (23.9)	310 (18.3)	
Investor-owned	214 (19.1)	181 (10.7)	
**Location**			0.1915
Urban	671 (59.9)	1053 (62.3)	
Rural	450 (40.1)	637 (37.7)	
**Teaching Status**			<.0001
Major	57 (5.1)	166 (9.8)	
Minor	263 (23.5)	449 (26.6)	
Non-teaching	801 (71.5)	1075 (63.6)	
**Inpatient Bed Capacity**			<.0001
500 or more beds	64 (5.7)	176 (10.4)	
400-499 beds	59 (5.3)	85 (5)	
300-399 beds	100 (8.9)	163 (9.6)	
200-299 beds	143 (12.8)	238 (14.1)	
100-199 beds	228 (20.3)	389 (23)	
<100 beds	527 (47)	639 (37.8)	
**Geographical Region**			<.0001
South Atlantic	1	274 (16.2)	
East North Central	0	304 (18)	
Middle Atlantic	0	195 (11.5)	
West South Central	197 (17.6)	198 (11.7)	
Pacific	150 (13.4)	150 (8.9)	
West North Central	176 (15.7)	220 (13)	
Mountain	133 (11.9)	133 (7.9)	
New England	0	91 (5.4)	
East South Central	1	125 (7.4)	
**Medical School Affiliation**			<.0001
Yes	295 (26.3)	562 (33.3)	
