# Supplementary material for: Gaps in Emergency General Surgery Coverage in the United States
Source: Ann Surg Open. 2021 Feb 18;2(1):e043. doi: 10.1097/AS9.0000000000000043 (PMC8409136; doi:10.1097/AS9.0000000000000043)
Supplement: Supplementary file 3 [file as9-2-e043-s003.pdf]

Appendix 3

Supplemental Table - Characteristics and Operating Room Access Variables among acute care general hospitals in the United States that do not provide round-the-clock emergency general surgery (EGS) care by percentage of time round-the-clock care is lacking (n=279)

Variable		1-20% not providing round-the-clock EGS n (%)	>20% not providing round-the-clock EGS n (%)	41-60% not providing round-the-clock EGS n (%)
**Hospital Characteristics**				
**Ownership**				
	Non-governmental	75 (60.0%)	60 (47.6%)	0.1106
	Governmental (non-federal)	34 (27.2%)	49 (38.9%)	
	Investor-owned	16 (12.8%)	17 (13.5%)	
**Location**				0.2792
	Urban	72 (57.6%)	64 (50.8%)	
	Rural	53 (42.4%)	62 (49.2%)	
**Teaching status**				
	Major	-	1 (0.8)	0.4088
	Minor	21 (16.8%)	16 (12.7%)	
	Non-teaching	104 (83.2%)	109 (86.5%)	
**Medical school affiliation**				
	Yes	14 (11.2%)	12 (9.5%)	0.6630
	No	111 (88.8%)	114 (90.5%)	
**Bed size**				
	<199	120 (96.0%)	123 (97.6%)	0.3137
	200-499	5 (4.0%)	2 (1.6%)	
	500 or more beds	-	1 (0.8%)	
**Region**				
	New England	5 (4.0%)	1 (0.8%)	0.3042
	East North Central	26 (20.8%)	18 (14.3%)	
	East South Central	14 (11.2%)	14 (11.1%)	
	Middle Atlantic	4 (3.2%)	9 (7.1%)	
	Mountain	15 (12.0%)	15 (11.9%)	
	Pacific	6 (4.8%)	5 (4.0%)	
	South Atlantic	12 (9.6%)	12 (9.5%)	
	West North Central	30 (24.0%)	28 (22.2%)	
	West South Central	30 (24.0%)	28 (22.2%)	
**Trauma Certification**				
	Yes	51 (42.5%)	52 (44.4%)	0.7627
	No	69 (57.5%)	65 (55.6%)	
**Operating Room Availability**				

Number of operating rooms			
<10	111 (94.1%)	111 (98.2%)	0.0117
10-20	7 (5.9%)	-	
>20	-	2 (1.8)	
Block Time for EGS (%)			
<1 Block time	114 (91.9%)	115 (95.8%)	0.4153
1-4 Days	7 (5.7%)	4 (3.3%)	
>5 days	3 (2.4%)	1 (0.8%)	
Tiered system for booking emergent surgical cases<br>Yes<br>No	 <br>67 (58.8%)<br>47 (41.2%)	 <br>33 (29.0%)<br>81 (71.1%)	 <br><0.0001
Process to defer elective cases<br>Yes<br>No	 <br>81 (70.4%)<br>34 (29.6%)	 <br>58 (50.9%)<br>56 (49.1%)	 0.0024
**Surgical Coverage**			
Daytime surgeons covering EGS free of other clinical duties<br>Yes<br>No	 <br><br>2 (1.6%)<br>122 (98.4%)	 <br><br>1 (0.9%)<br>111 (99.1%)	 0.6220
Daytime surgeon on call for EGS working post-call			0.3294
Always/Often	118 (97.5%)	101 (98.1%)	
Sometimes	2 (1.7%)	-	
Rarely/Never	1 (0.8%)	2 (1.9%)	
In-house surgeon overnight for EGS			0.0515
Always/Often	31 (25.6%)	15 (14.6%)	
Sometimes	6 (5.0%)	11 (10.7%)	
Rarely/Never	84 (69.4%)	77 (74.8%)	
Overnight surgeon also responsible for covering trauma			0.0007
Always/Often	80 (66.1%)	45 (43.7%)	
Sometimes	4 (3.3%)	14 (13.6%)	
Rarely/Never	37 (30.6%)	44 (42.7%)	
Overnight surgeon also responsible for covering ICU care			0.0486
Always/Often	34 (28.1%)	17 (16.4%)	
Sometimes	11 (9.1%)	6 (5.8%)	
Rarely/Never	76 (62.8%)	81 (77.9%)	

Overnight surgeon also responsible for covering EGS at more than one hospital			0.0155
Always/Often	14 (11.6%)	24 (23.5%)	
Sometimes	17 (14.1%)	6 (5.9%)	
Rarely/Never	90 (74.4%)	72 (70.6%)	
Surgeon Covering EGS receives stipend beyond billing for services rendered			0.0130
Always/Often	23 (19.0%)	8 (7.7%)	
Sometimes	10 (8.3%)	4 (3.9%)	
Rarely/Never	88 (72.7%)	92 (88.5%)	
**Overnight Peri-Operative Staffing**			
Overnight scrub techs			
None	-	1 (1.4%)	0.4128
On-call	108 (98.2%)	68 (95.8%)	
In-house	2 (1.8%)	2 (2.8%)	
Overnight OR nurses			
None	-	-	0.3263
On-call	109 (99.1%)	69 (97.2%)	
In-house	1 (0.9%)	2 (2.8%)	
Overnight recovery room nurses			0.7205
None	6 (5.5%)	4 (5.6%)	
On-call	102 (93.6%)	67 (94.4%)	
In-house	1 (0.9%)	-	
Overnight anesthesia staff (MD, DO, CRNA)			
None	70 (66.7%)	45 (70.3%)	0.3885
On-call	32 (30.5%)	15 (23.4%)	
In-house	3 (2.9%)	4 (6.3%)	
*Student T-test, Wilcox Rank Sum and Chi^2 tests of association where appropriate			
